# Walking the tightrope: UCH-L1 as an mTOR inhibitor and B-cell oncogene

**DOI:** 10.18632/oncotarget.27154

**Published:** 2019-08-27

**Authors:** Tibor Bedekovics, Sajjad Hussain, Paul J. Galardy

**Affiliations:** Department of Pediatric and Adolescent Medicine, Mayo Clinic, Rochester, MN, USA; Division of Pediatric Hematology-Oncology, Mayo Clinic, Rochester, MN, USA

**Keywords:** lymphoma, animal model, protein synthesis

It is clear that ubiquitination is about far more than regulating when and where a protein is degraded. The localization, activity, and binding partners of proteins is affected by the addition of ubiquitin. Necessarily, the impact of this modification depends on the rate of ubiquitin addition and removal. There are nearly 100 de-ubiquitinating enzymes encoded in the human genome, the functions of which are relatively unstudied. The enzyme UCH-L1 (*UCHL1*; also known as PGP9.5) was one of the first discovered – yet remains enigmatic. Several reports have drawn connections between UCH-L1 and cancer – both as an oncogene and as a tumor suppressor. Recently, we have found new mechanistic insight into the activity of UCH-L1 may suggest how this one protein might do both [1].

UCH-L1 was discovered in 1981 by two-dimensional protein electrophoresis in human brain and designated as Protein Gene Product 9.5 (PGP9.5) based on its migration distance [2]. After being found to possess ubiquitin hydrolase activity, a small subfamily of four ubiquitin carboxy-terminal hydrolases were defined from calf-thymus, with the “L1” enzyme representing the smallest member resolved by gel filtration. Encoded on chromosome 4p13, the *UCHL1* gene product is a 25 kDa protein that localizes to the cytosol and nucleus. It is conserved through all vertebrates and is expressed predominantly in neuro-endocrine tissues. It was unexpected then that UCH-L1 was found to be strongly expressed in Burkitt lymphoma cell lines, and its expression induced in primary human B-cells after transduction with the Epstein-Barr virus [3]. Whether its production in these tumors was a cause or consequence of the malignant state was unknown.

To clarify its role in cancer we generated transgenic mice overexpressing UCH-L1 (*Uchl1*^Tg^) from a ubiquitous promoter, driving expression in most tissues [4]. These mice developed spontaneous lymphomas and lung tumors, providing evidence that UCH-L1 is a potent oncogene. The lymphomas are B-cell in origin and their development is accelerated when *Uchl1^Tg^* mice are crossed either with the Eμ-myc or Iμ-HABCL6 models [4, 5]. As typically the genes that contribute to B-cell lymphomagenesis also play a role in physiological events in B-cell development, we were curious as to why a neuron-specific enzyme would have oncogenic activity in B-cells. While bulk gene expression studies do not show expression of *UCHL1* in lymphoid tissues, a re-evaluation of published data from purified B-cell subsets showed a strong induction in germinal-center centroblasts and centrocytes [5]. We confirmed this by immunohistochemistry, with no substantial polarization between the light or dark-zones of the germinal center. Whether UCH-L1 plays a role in the germinal center reaction continues to be a subject of study.

At the molecular level, the function of UCH-L1 has been difficult to define. After finding an increased incidence of lymphoma in *Uchl1*^Tg^ mice, we used a candidate approach to find signaling pathways deregulated by UCH-L1. We found that UCH-L1 stimulated phosphorylation of the pro-survival kinase AKT, particularly at the hydrophobic motif (AKT^S473^) [4]. We have subsequently found that the mechanism stems from a direct effect on the mTOR kinase complexes, one of which catalyzes the phosphorylation of AKT^S473^. UCH-L1 expression does not change the level of PDK1 (the kinase responsible for p-AKT^T308^) or mTOR (responsible for p-AKT^S473^), but tips the balance of the relative amounts of mTOR complexes [6]. In malignant and primary B-cells, as well as in the nervous system, UCH-L1 increases the levels of the AKT phosphorylating mTOR complex 2 while suppressing the levels of the rapamycin-sensitive complex 1 that drives cap-dependent mRNA translation. The magnitude of the inhibitory effect on mTORC1 is similar to treating cells to rapamycin itself.

How then can an enzyme that mimics the activity of an anti-neoplastic drug drive the development of cancer? To answer this, we used a proximity-based proteomics approach to identify other proteins that associate with, or are substrates for, UCH-L1 [1]. Through this approach, we recently identified the association of UCH-L1 with the translation initiation complex eIF4F – the assembly of which is under the control of mTORC1. Bypassing the need for mTORC1 activity, UCH-L1 itself promotes the assembly of elF4F. Consistent with this, protein synthesis is preserved and even increased in cells expressing UCH-L1 – despite reduced mTORC1 activity. While this has helped to explain how an mTOR inhibitor promotes cancer, there are many unanswered questions. Why do some tissues respond to increased UCH-L1 activity by developing cancer, whereas others do not? Perhaps in tissues that are ‘resistant’ to the oncogenic effects of UCH-L1 (e.g. brain, adrenal medulla) there are downstream mechanisms to mitigate the higher AKT signaling and eIF4F assembly. If UCH-L1 truly has tumor suppressor activity in certain tissues, one might imagine that the higher AKT signaling in these cellular contexts more easily drive oncogene induced senescence. Finally, the physiological importance of these biochemical effects in the germinal center are poorly understood. Ongoing work seeks to unravel these and other questions as we continue to understand this enigmatic enzyme.
